# A long-lasting low oxygen saturation hiding Titusville hemoglobin diagnosis in a premature 14-month-old child – case report

**DOI:** 10.1186/s13052-025-01900-4

**Published:** 2025-03-23

**Authors:** Maria Francesca Patria, Marta Piotto, Cristina Curcio, Silvana Gangi, Youssra Belhaj, Mara Lelii, Barbara Madini, Alessia Rocchi

**Affiliations:** 1 Fondazione IRCCS Ca’ Granda, Ospedale Maggiore Policlinico, Pediatria Pneumoinfettivologia, 20122 Milan, Italy; 2 Università degli Studi di Milano, 20122 Milan, Italy; 3 Fondazione IRCCS Ca’ Granda, Ospedale Maggiore Policlinico, Medical Genetics Laboratory, 20122 Milan, Italy; 4 Fondazione IRCCS Ca’ Granda, Ospedale Maggiore Policlinico, Neonatal Intensive Care Unit (NICU), 20122 Milan, Italy

**Keywords:** Hypoxemia, Hb Titusville, Oxygen therapy, Hemoglobinopathy, Bronchopulmonary dysplasia

## Abstract

**Background:**

There are some clinical conditions that can impact the accuracy of spO_2_ measurements, leading to an incorrect diagnosis of hypoxemia. Low oxygen affinity hemoglobinopathy can present with low spO_2_ and discordance with PaO_2_ and general clinical conditions.

**Case Presentation:**

We report the case of a 14-month-old male, born extremely premature, with severe bronchopulmonary dysplasia (BPD). He required continuous high-flow nasal cannula (HFNC) oxygen therapy and was hospitalized due to a viral respiratory infection causing dyspnea and desaturation. Once the child clinically recovered, all attempts to wean him off oxygen resulted in rapid drops in SpO2. This occurred despite the absence of increased work of breathing, cyanosis, or abnormal PaO2.

**Conclusions:**

Severe BPD and recurrent respiratory issues could have per se justified the persistently low spO2. Incongruence between low spO_2_ values, normal PaO_2_ levels and good clinical condition, once the child was recovered, raised suspicion of low oxygen affinity hemoglobin (Hb) variants. Abnormal Hb peak detected through high-performance liquid chromatography allowed rare diagnosis of Hb Titusville, confirmed by molecular analysis. In conclusion, the case matched a rare low oxygen affinity hemoglobinopathy (Hb Titusville), highlighting its relevance in unexplained hypoxemia. Diagnosis challenges necessitate a systematic approach to prevent misinterpretations.

## Background

Oxygen saturation measurement by pulse oximeter (spO_2_) is a non invasive, indirect method to estimate the level of oxygenated hemoglobin (Hb). Pulse oximetry has become a common practice in many clinical contexts, including the Intensive Care setting, given the good correlation with the arterial partial pressure of oxygen (PaO_2_), an invasive measurement of the actual arterial oxygen content. However, there could be some clinical conditions that can impact the accuracy of spO_2_ measurements, leading to an incorrect diagnosis of hypoxemia. Hemoglobins with low oxygen affinity are a rare group of variant Hb, which shift the oxygen-Hb dissociation curve to the right and can present with low spO_2_ and discordance with normal PaO_2_. A concurrent respiratory disease, as well as bronchopulmonary dysplasia of the preterm, might make it difficult to reach a clinical suspicion.

## Case presentation

A 14-month-old male, born extremely premature (26 weeks gestation), presenting severe bronchopulmonary dysplasia (BPD) that still required continuous high-flow nasal canula (HFNC) oxygen therapy (FiO_2_ 0.30) at his age, was hospitalized for dyspnea, desaturation (spO_2_ 86% in HFNC, FiO2 0.30) and insufficient response to increased amount of oxygen (spO2 93% in HFNC, FiO2 0.45). His medical history was remarkable for recurrent respiratory exacerbations, which frequently led to hospitalization and transient increase in oxygen level administered.

**Fig. 1:**
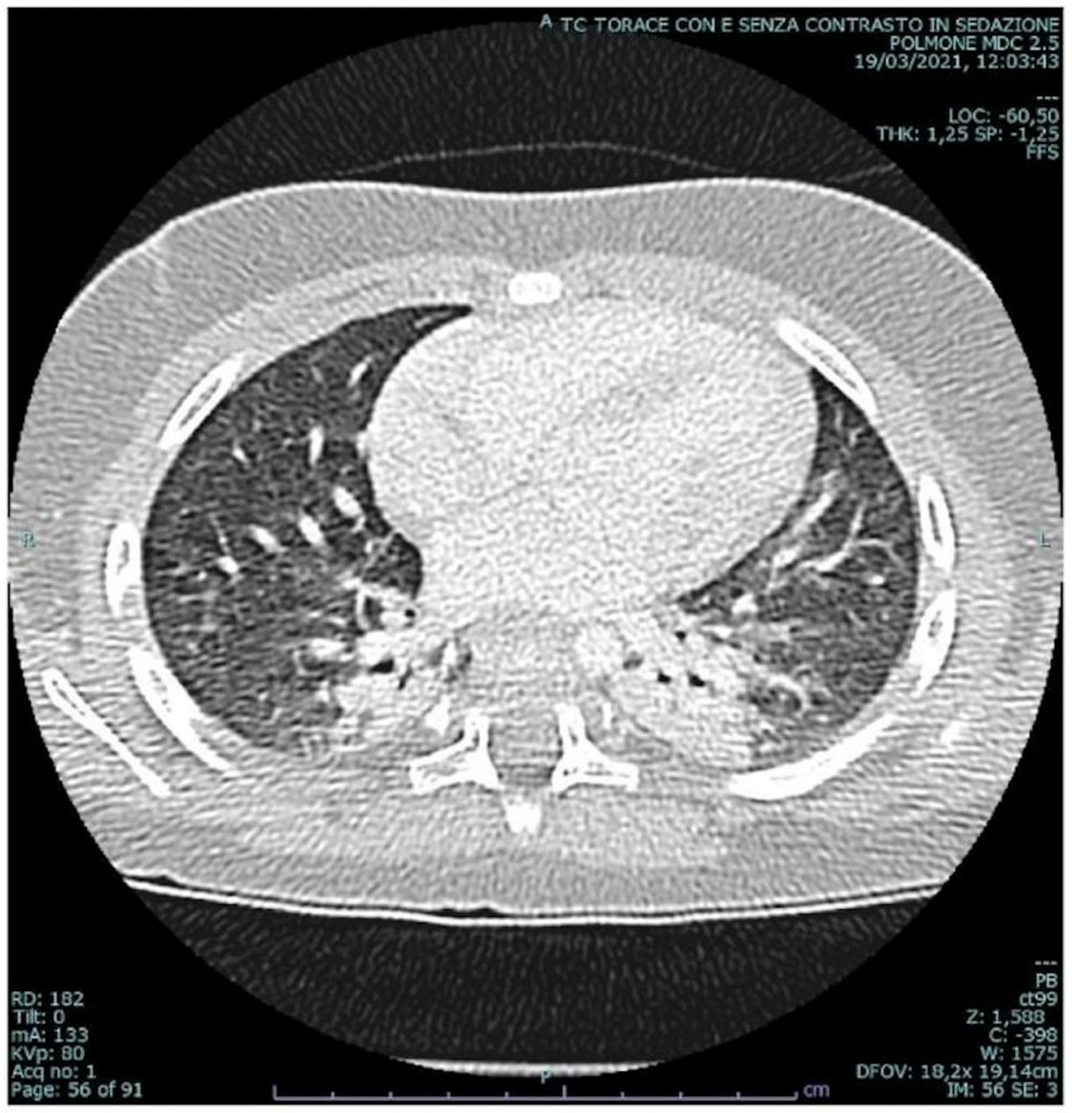
High resolution CT bilateral consolidations in the apical segment of the lower lobes with air bronchogram

**Fig. 2:**
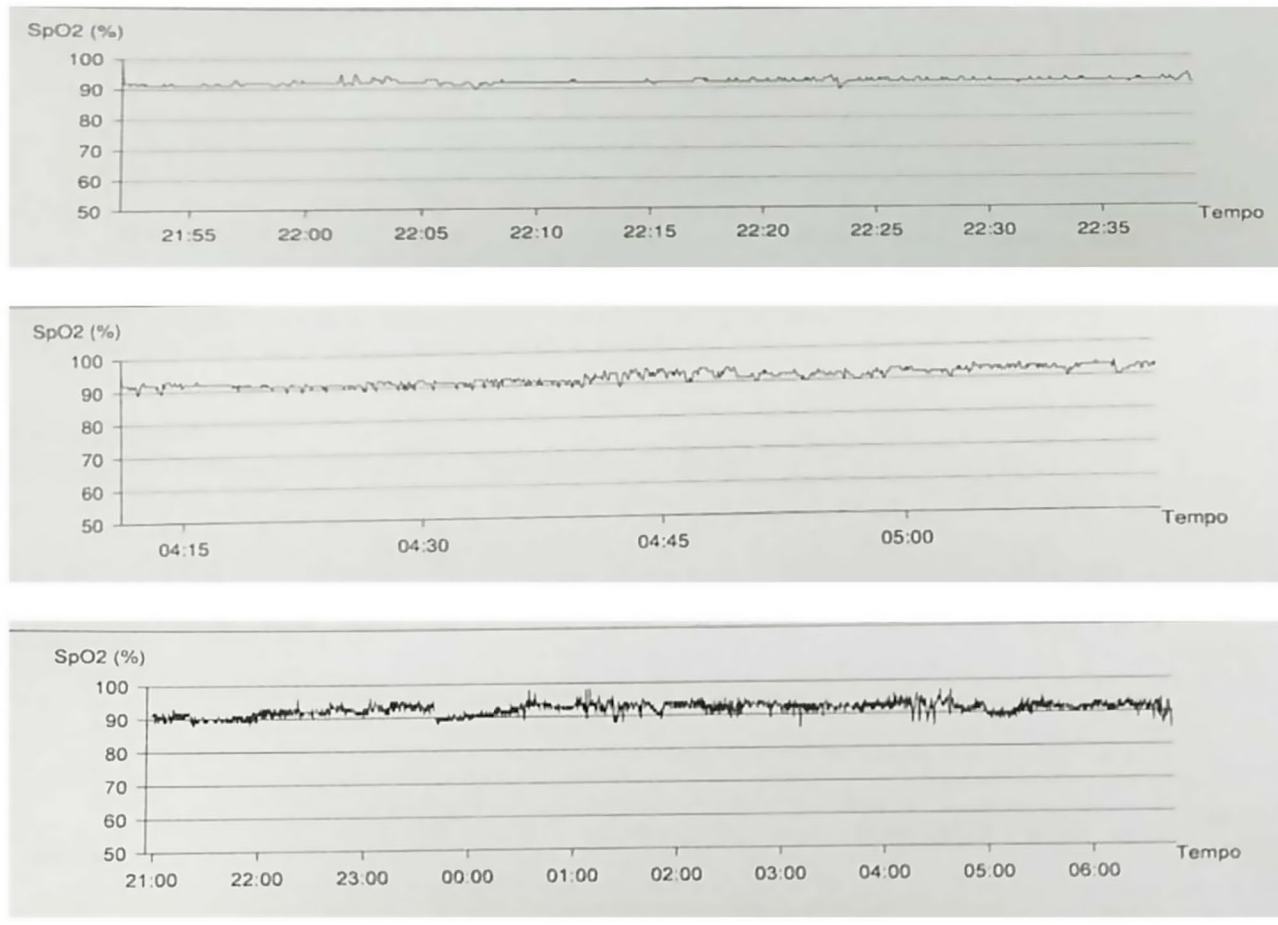
Nocturnal pulse oximetry measurement on oxygen supplementation (FiO2 0.30) through different respiratory supports. Top to bottom: High-flow nasal canula: 2 L/Kg; CPAP 8 cm H20; Bipap: IPAP: 10 cm H2O, EPAP: 5 cm H2O

Human rhinovirus was detected in the nasopharyngeal aspirate and his blood count showed Hb 11.3 g/dl, white blood cells 17,900 × 10^9^/L, and C-reactive protein 2.63 mg/dl. The child was successfully treated with nebulized albuterol, intravenous steroids and increased oxygen supplementation (FiO_2_ up to 0.45). On day 5 he fully recovered (no pathologic sounds on auscultation, respiratory rate 32 breaths/min, pulse 115 bpm, blood pressure 97/53 mmHg) and spO_2_ returned to pre-exacerbation levels (95% on HFNC, FiO_2_ 0.30). However, attempts of oxygen weaning always led to a rapid drop to spO_2_ of 85–88%, though without increased work of breathing, pulse rate or cyanosis. A contrast-enhanced chest CT scan detected the presence of consolidations with aerial bronchogram in the paravertebral portions of the lower lobes (Fig. 1); echocardiographic assessment resulted normal, and the arterial blood gas (ABG) on room air revealed respiratory alkalosis (pH 7.54, PaCO_2_ 26 mmHg, PaO_2_ 114 mmHg, HCO_3_ 25.8 mmol/L). SpO2 monitoring, conducted over three separate nights with different respiratory supports (HFNC, CPAP, and BiPAP, respectively) on oxygen supplementation, showed a mean SpO2 value of 92%, regardless of the airway pressure used (Fig. 2). Nocturnal transcutaneous carbon dioxide monitoring (tCO_2_) was normal (mean value: 38 mmHg, maximum value: 43 mmHg). Incongruence between low spO2 values, normal ABG PaO2 levels and an overall good clinical condition has raised the suspicion of low oxygen affinity variant Hb.

## Discussion and conclusion

Hypoxemia is defined as a reduction of oxygen tension in the arterial blood. The main underlying mechanisms of hypoxemia are mostly related to ventilation/perfusion (V/Q) mismatch, right-to-left shunt, diffusion impairment or hypoventilation [1]. The CT scan revealed lung atelectasis with low ventilation/perfusion (V/Q) ratios, which could have contributed to hypoxemia without CO2 retention. In such cases, ventilatory support with positive end-expiratory pressure (CPAP or BiPAP) typically improves alveolar recruitment [2] and increases SpO2. However, we did not observe any improvement in SpO2 with these interventions.

BPD is the most common complication of prematurity, characterized by a multifactorial disruption of alveolar and vascular development and subsequent chronic sequelae. Approximately one-quarter of extremely preterm BPD infants are discharged with supplemental oxygen to minimize lung injury and promote growth and neurodevelopment [3]. Long-term oxygen therapy may be required for variable duration, the median age of oxygen weaning ranging from 10 to 15 months [4]. Therefore, the requirement of a full-day and continuous oxygen supplementation in a 14-month-old, well grown child, clinically asymptomatic once recovered from the rhinovirus infection seemed quite unusual.

Finally, the normality of echocardiographic findings and the physiological range of nocturnal tCO_2_, allowed to reasonably rule out cardiovascular alterations, diffusion impairment and hypoventilation.

SpO_2_ detected by pulse oximetry measures the ratio between oxygenated and deoxygenated Hb, while PaO_2_ provides the partial pressure of oxygen dissolved in the arterial blood. In physiological conditions there is an optimal correlation between SpO_2_ and PaO_2_, and a decrease in SpO_2_ is a reliable sign of hypoxemia. Mutations in the Hb α and/or β chains can alter the oxygen-carrying affinity of hemoglobin. In low-affinity Hb variants, the sigmoidal oxygen-Hb dissociation curve shifts to the right. This reduces the binding of oxygen to Hb but enhances oxygen release at higher partial pressures, improving cellular oxygen availability [5]. In this condition, spO_2_ may be significantly lower than PaO_2,_ and P_50,_ a blood gas parameter which represent the oxygen tension at 50% saturation and measures Hb-oxygen affinity, is increased [6]. Unfortunately, P_50_ was not routinely calculated by blood gas analyzers and this incomplete information may have contributed to the initial misinterpretation of the ABG results. In addition, respiratory alkalosis was another indirect sign of normal gas exchange in the lungs, as the low levels of carbon dioxide detected were likely related to a relative hyperventilation due to an unnecessary respiratory support.

**Fig. 3:**
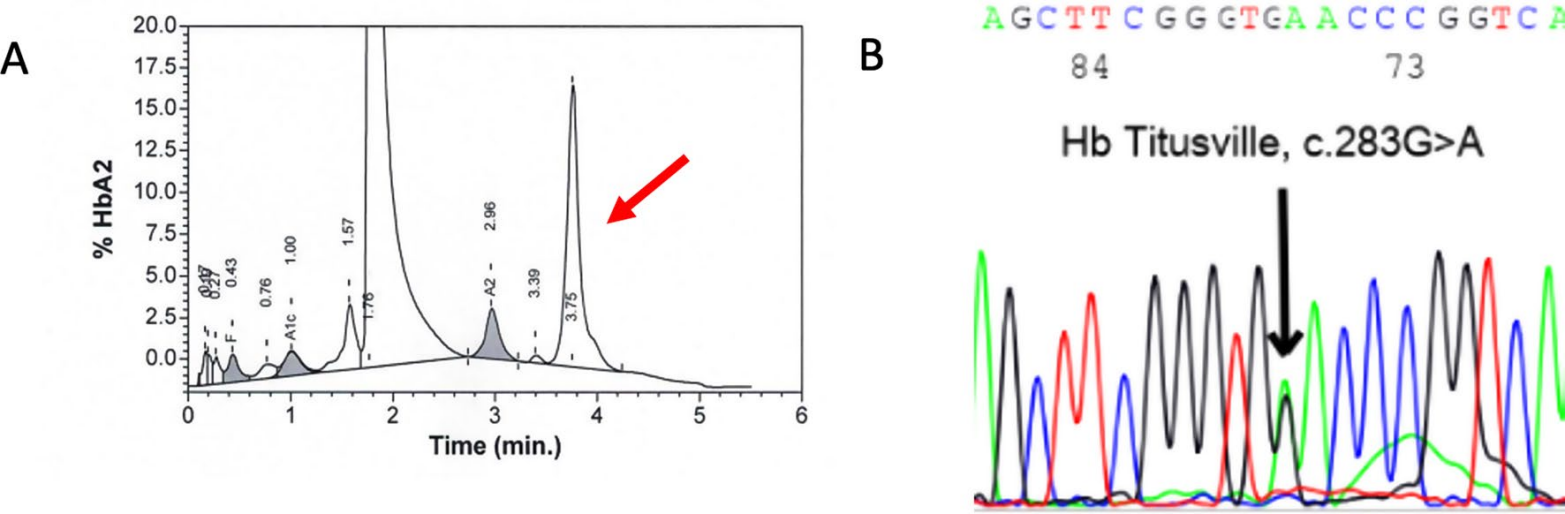
**A** High-performance liquid chromatographic showing an abnormal Hb peak (arrow). **B** Gene sequencing (electropherogram) showing codon 94 GAC > AAC mutation in alpha 1 gene

The final investigation included a high-performance liquid chromatography, which led to an abnormal Hb peak (15.9% Hb G Philadelphia?); further molecular analysis have, instead, detected a heterozygous mutation for c.283G > A in the HBA1 gene, consistent with Hb Titusville, a rare low-oxygen affinity Hb variant (Fig. 3). Moreover, the father of the child exhibited an asymptomatic spO2 of 85% in room air and the Hb analysis of relatives documented the same mutation both in the patient’s father and paternal uncle (all of Bangladeshi origin). Since its first description in 1975, Hb Titusville has only been reported in about 20 cases, often in the same family, among different ethnicities. Affected individuals are mostly asymptomatic, except for eventual mild anemia and exertion dyspnea. This condition does not require any specific management for hypoxia and no long-term consequences are reported, not even in case of general anesthesia or major surgeries [7]. The diagnosis may however be challenging when dealing with comorbidities, as in this case; nevertheless a rational diagnostic approach is crucial in order to avoid misdiagnoses and therapeutic mistakes.

## Data Availability

The complete data reported in the current case report are available from the corresponding author on reasonable request.

## Abbreviations

BPD: Bronchopulmonary Dysplasia
HFNC: High-Flow Nasal Cannula
FiO2: Fraction of Inspired Oxygen
spO2: Oxygen Saturation
Hb: Hemoglobin
ABG: Arterial Blood Gas
CT: Computed Tomography
CPAP: Continuous Positive Airway Pressure
BiPAP: Bilevel Positive Airway Pressure
PaCO2: Partial Pressure of Carbon Dioxide in Arterial Blood
PaO2: Partial Pressure of Oxygen in Arterial Blood
HCO3: Bicarbonate
tCO2: Transcutaneous Carbon Dioxide
